# Experiences of quitting smoking in prisons: A qualitative study of people in custody

**DOI:** 10.18332/tid/183604

**Published:** 2024-02-19

**Authors:** Xue Weng, Emily Ching Ma, Chu Yu Song, Jung Jae Lee, Henry Sau Chai Tong, Vienna Wai Yin Lai, Tai Hing Lam, Man Ping Wang

**Affiliations:** 1 Institute of Advanced Studies in Humanities and Social Sciences, Beijing Normal University, Zhuhai, China; 2 School of Nursing, Li Ka Shing Faculty of Medicine, the University of Hong Kong, Hong Kong SAR, China; 3 School of Public Health, Brown University, United States; 4 School of Social Development and Public Policy, Beijing Normal University, Beijing, China; 5 Hong Kong Council on Smoking and Health, Hong Kong SAR, China; 6 School of Public Health, Li Ka Shing Faculty of Medicine, the University of Hong Kong, Hong Kong SAR, China

**Keywords:** prison, people in custody, quitting, qualitative study

## Abstract

**INTRODUCTION:**

Smoking prevalence among people in custody (PIC) is extremely high, and prison-based smoking cessation interventions are needed. The study explored the quitting experiences of PIC who participated in the ‘Quit to Win’ contest (QTW).

**METHODS:**

This qualitative study, conducted from 2019 to 2021 in two Hong Kong prisons, included semi-structured individual interviews with 26 PIC (13 men and 13 women) who were participants in QTW and two correctional staff who coordinated QTW. A semi-structured interview guide with open-ended questions was developed to examine multilevel factors that promote or impede smoking cessation in prisons. Maximum variation sampling was used to ensure a diverse range of social, demographic, and smoking profiles. Data were managed and analyzed using thematic analysis.

**RESULTS:**

Two themes were identified from the data: 1) quitting in prison: barriers and facilitators; and 2) QTW in prison: a trigger for behavior change. Barriers (i.e. stress, boredom, isolation, lack of self-autonomy, nicotine dependence and lack of cessation medication, barriers to moving to a different wing) and facilitators (i.e. concerns about health, money savings, and the smoke-free wing) that impeded or supported smoking cessation during incarceration were identified. QTW provided health education, quitting incentives, and social support that helped PIC overcome the barriers of quitting by serving as a trigger for behavior change. Notably, social visits with family were identified as key drivers of PIC’s quitting success, whereas their suspension during the COVID-19 pandemic disincentivized their abstinence.

**CONCLUSIONS:**

This study introduced the QTW contest to prisons and provided qualitative evidence on the multilevel factors promoting or impeding smoking cessation in prison. QTW helped PIC overcome the barriers of quitting by serving as a trigger for behavior change. Future prison-based interventions should leverage social support, enhance stress-coping skills, facilitate access to pharmacotherapy, and collaborate with correctional services agencies.

## INTRODUCTION

Despite declining smoking prevalence in the general population in most developed countries^1^, smoking prevalence among people in custody (PIC; people on remand awaiting trial, awaiting sentencing, or serving a custodial sentence) is extremely high^2^. Over 14.5 million smokers are in custody worldwide, and smoking rates are notably higher than those in the general population^2^. Specifically, the prevalence is 1.3- to 7.3-fold higher for male inmates and 1.7- to 62.6-fold higher for female inmates^2^. The high smoking prevalence is because PIC are drawn disproportionately from marginalized and socially disadvantaged populations, such as groups with low socioeconomic status^3^, individuals with mental illness^4^, and illicit drug use^5^. Moreover, incarcerated smokers bear a heavier burden of smoking-related disease and mortality than the general population^6,7^.

Although smoking bans in prisons have been introduced in many developed countries, relapse rates are high (>60%) after release from smoke-free prisons^8^. In Hong Kong, although smoking is not permitted in smoke-free wings (i.e. detention areas) of prisons, PIC in smoking wings are allowed to smoke in their cells and designated outdoor areas. Previous studies on correctional settings have reported mainly on the resumption of smoking after release from smoke-free prisons^8,9^, and the experiences of quitting in prisons where smoking is still permitted have rarely been reported. An in-depth understanding of the quitting experiences of PIC would inform the development of more effective smoking cessation interventions that specifically target this high-risk group.

‘Quit and Win’ contests are interventions that use potential rewards to motivate smokers to quit smoking^10^. The prize provides an additional incentive for smokers to start and maintain quit attempts. Our previous trials showed that this intervention was effective in promoting smoking cessation in the general population^11-14^. However, our literature search in PubMed (up to November 2023) using the keywords ‘prison’, ‘incarceration’, ‘custody’, ‘smoking’, ‘quitting’, and ‘contest’ found that no study has conducted a smoking cessation contest in prison or explored the quitting experiences of PIC during incarceration while participating in a smoking cessation contest.

This qualitative study aimed to describe the quitting experiences of PIC who participated in the smoking cessation contest to inform future interventions to address smoking-related health disparities in prisons. We explored multilevel factors (i.e. individual, social, and institutional factors) that affected smoking cessation in prisons and the role of the smoking cessation contest in their quitting process.

## METHODS

### Study design and setting

We conducted a qualitative study with PIC participating in the ‘Quit to Win’ contest (QTW), an annual smoking cessation campaign organized by the Hong Kong Council on Smoking and Health since 2009 to recruit smokers in community settings^11-14^. Since 2018, QTW has been introduced to prisons in collaboration with the Correctional Services Department^15^. During 2018–2021, 59 PIC from two prisons (one male and one female prison) were enrolled in QTW. This program included interventions (e.g. cessation advice and courses) with follow-up at 1, 2, 3, and 6 months. QTW participants who successfully quit during the contest, with biochemical validation at 3 and 6 months, received up to HK$1000 (about US$125). After the 6-month follow-up, QTW participants were invited to individual interviews about their quitting experience. Ethical approval was granted by the Institutional Review Board of the University of Hong Kong/Hospital Authority Hong Kong West Cluster, and permissions were obtained from the prison authorities. The participants provided written informed consent and were informed that they could withdraw from participation at any time during the interview.

### Participants

PIC were eligible to participate in QTW if they were Hong Kong residents aged ≥18 years; daily smokers in prisons, with their status validated by exhaled CO (≥4 parts per million) or salivary cotinine (≥10 ng/mL) tests; able to communicate in Cantonese or read Chinese; and motivated to quit or reduce smoking.

We used maximum variation sampling (a type of purposive sampling)^16^ to recruit QTW participants with diverse social demographics and smoking profiles in order to ensure an adequate breadth of narratives for the analysis. A total of 26 PIC, including 14 PIC (seven men and seven women) in April–May 2019 and 12 PIC (six men and six women) in March–April 2021, were recruited for and completed the interviews. We also interviewed two correctional staff members (one male staff member from the male prison and one female staff member from the female prison) who coordinated QTW to explore their experience in providing the quitting service to PIC.

### Data collection

Thirty-minute individual semi-structured interviews (face-to-face) were conducted in meeting rooms in the two prisons. An interview guide with open-ended questions (Table 1) was used to ask about the participants’ perceptions of smoking and their quitting experience, including facilitators or barriers to sustained abstinence during incarceration. Data saturation was reached after 23 interviews, with three additional interviews confirming that no new information emerged. The interviews were conducted by two trained research staff with previous experience in health promotion and qualitative research. One research staff member (interviewer) asked the questions, and the other staff member (scribe) wrote down the key answers from participants, as audio recordings were not allowed in the prisons. A senior researcher supervised the data collection and held regular feedback sessions with the interview staff shortly after the interviews to obtain their reflexivity regarding the interviews and data collection process^17^.

**Table 1:** Interview guide for the qualitative study, conducted among people in custody (PIC) in two Hong Kong prisons, 2019–2021

***1. Interview guide to PIC***
**Smoking experience** Can you describe your smoking experiences in prison? How do you get access to cigarettes in prison? What are the main factors that motivate you to smoke in prison? **Quitting experience** Have you ever attempted to quit smoking in prison before joining the Quit to Win (QTW) program? How did you hear about QTW? What motivated you to participate in the program? In what ways has QTW helped you quit or reduce smoking? Did the correctional staff or your quitting buddy provide support during your quitting process? What were the main barriers you faced during your quitting process? Do you have any suggestions to improve QTW?
***2. Interview guide to correctional staff***
Can you share your observations on the smoking prevalence among PIC? Based on your experience, what motivates PIC to quit smoking? Did QTW helped PIC quit or reduce smoking? What kinds of techniques or strategies did you find to be most effective? Based on your working experiences, what are the unique challenges that PIC face when trying to quit smoking compared to individuals in the general population? Do you have any suggestions to help PIC quit smoking in prisons?

### Data analysis

A thematic analysis approach developed by Braun and Clarke^18^ was used to analyze the interview data. Two members of the research team (XW and EM) led the data analysis, with additional coding and analytic support from other members. During the initial phase, the two lead analysts conducted line-by-line coding to familiarize themselves with the raw data and identify recurring patterns. After extensive rereading of the data, codes were labeled under broader concepts, which were then clustered into major themes. Discrepancies in coding were resolved by discussion with the research team.

The interview transcripts from the scribes were analyzed in Cantonese. Relevant quotes were translated into English to illustrate key findings and then back-translated for assessment by bilingual researchers (Cantonese and English). The rigor of this study was examined in four domains: epistemological integrity, analytic logic, representative credibility, and interpretive authority^19^.

## RESULTS

### PIC participants’ demographics

Table 2 shows that half of the 26 PIC participants were male, aged 30–49 years, and married. Twenty reported illicit drug use prior to incarceration, and ten were serving sentences of 5–10 years, and ten for >5 years. Seventeen participants had started smoking younger than 18 years, 17 were ready to quit within 30 days at baseline, and 22 had quit smoking at the time of the interview (i.e. 6-month follow-up).

**Table 2:** Characteristics of participants in the qualitative study, from two Hong Kong prisons, 2019–2021 (N=26)

*Characteristics*	*n (%)^a^*
**Sex**	
Male	13 (50.0)
Female	13 (50.0)
**Age** (years)	
≤29	11 (42.3)
30–49	13 (50.0)
≥50	2 (7.7)
**Marital status**	
Single	11 (42.3)
Married/cohabiting	13 (50.0)
Divorced/separated/widowed	2 (7.7)
**Have children**	
Yes	9 (36.0)
No	16 (64.0)
**History of illicit drug use**	20 (80.0)
**Sentence length** (years)	
<5	2 (9.1)
5–10	10 (45.5)
>10	10 (45.4)
**Cigarette consumption changes after imprisonment**	
Unchanged	2 (7.7)
Increased	23 (88.5)
Decreased	1 (3.8)
**Age starting smoking** (years)	
≤17	17 (68.0)
18–25	7 (28.0)
≥26	1 (4.0)
**Readiness to quit at baseline** (days)	
≤30	9 (34.6)
>30	17 (65.4)
**Reasons for joining QTW^b^**	
Concern about health	19 (73.1)
Chances to see family members	18 (69.2)
Cigarettes are expensive	17 (65.4)
Becoming a role model for their children	8 (30.8)
To receive cash coupon	8 (30.8)
Suggested by health professionals	3 (11.5)
**Quitting status at the interview** (6-month follow-up)	
Quitters	22 (84.6)
Continuing smokers	4 (15.4)

QTW: ‘Quit to Win’ contest.

a Sample size varied because of missing data. Missing data are excluded.

b Participants could choose more than one answer.

**Table 3:** Quitting in prison: barriers and facilitators identified by this qualitative study of people in custody in two Hong Kong prisons

*Major themes*	*Selected quotations*
**Stress, boredom, isolation, and lack of self-autonomy**	*‘Quitting in prison is harder than quitting outside because [inside] there are few things to do for leisure, so I smoked for leisure.’* ( Female, quitter, ID1) *‘[I smoke because] I feel flustered, bored, and there is a lot to worry about.’* (Male, continuing smoker, ID14) *‘I did not smoke prior to [coming to] prison … Smoking was like a symbol of freedom to me. There were numerous rules inside that I had to follow. Smoking made me feel like I got some private time that I could break rules, which was relieving.’* (Male, quitter, ID17) *‘There is nothing to do to kill time.’* ( Male, quitter, ID18) *‘You cannot leave [a situation]… Outside, it is easier to not smoke because there are other things to do.’* (Female, quitter, ID21) *‘[Here,] there is nothing we can do… Every day is repetitive, the same work every day…’* ( Female, quitter, ID22) *‘I am truly unhappy [after imprisonment], so I smoke, but I know that smoking can’t solve the problem…’* (Female, continuing smoker, ID24) *Isolation during COVID-19:* *‘It would be better if there are more incentives like decreasing sentence length and extra visiting. After the cancellation of extra visiting and the award ceremony, the motivation for quitting is fairly reduced.’* ( Male, quitter, ID20) *‘We already don't have enough activities; there are fewer during the COVID outbreak.’* ( Female, quitter, ID22) *‘There is nothing much to do in prison, and PIC don’t know when they can leave. Since COVID-19 began, special visits from family have been cancelled. PIC smoke during COVID-19 because they worry that they are abandoned by their family and wonder if their family don’t disclose facts to them.’* ( Staff, ID27)
**Nicotine dependence and lack of cessation medication**	*‘I used to be addicted to alcohol, cocaine, and ketamine [before entering prison]. I got psychological cravings.’* (Female, quitter, ID5) *‘Rewards can be provided in the form of snacks, which are more appealing than cash coupons. That is because we cannot buy various kinds of snacks in prison. Scarce things are more precious.’* ( Male, quitter, ID11) *‘I tried nicotine patches for two weeks, and I felt confident to quit with it. However, no more patches were supplied after two weeks.’* (Male, continuing smoker, ID15) *‘I want to try nicotine patches. However, the procedures to get patches are complicated.’* ( Male, quitter, ID16) *‘I couldn't sleep well and felt easily exhausted after the first week of quitting.’* ( Male, quitter, ID19) *‘I hope there could be more welfare, such as a greater variety of snacks.’* (Female, quitter, ID22) *‘Many of the inmates started smoking at age 12–13 years. Some inmates have high nicotine dependence. They are willing to forgo two to three meals to smoke.’* ( Staff, ID27) *‘NRT is difficult to access. If needed, cessation medicines have to be prescribed by doctors based on the PIC's nicotine dependence. NRT patch doses are usually provided for up to two weeks' time and end afterward.’* ( Staff, ID27)
**Barriers to moving to a smoke-free wings**	*‘The [smoke-free] wing is not open for application yet, and I don't like the work there, so I prefer not to move.’* (Female, quitter, ID21) *‘If I were moved to a smoke-free wing, I must start over in a new job and at the lowest position.’* ( Female, quitter, ID23) *‘The assignment to a smoke-free and smoking wing is not decided by the inmate themselves upon admission. It is decided by the Correctional Services Department’s administration upon an exhaustive examination of their medical and psychosocial history, health condition, and the suitability of the jobs available in the wing.’* ( Staff, ID27) *‘Moving to a smoke-free wing is not too common merely for smoking cessation or relapse prevention due to practical concerns. They have to “live a new life” if they leave the original cells. Relationships and jobs have to start anew. They are major deterrents to the change in living space.’* ( Staff, ID28)
**Smoke-free wing**	*‘I want to apply to move to a smoke-free wing so that I will not relapse. In addition, the smoke-free wing gives us time to watch TV.’* ( Female, quitter, ID5) *‘Living and eating in the smoke-free wing are helpful. [There] you would not come into contact with cigarettes, which reduces temptation.’* ( Male, continuing smoker, ID14) *‘[In a smoke-free wing] it is much easier for me to quit smoking because the temptation is relatively small.’* (Male, quitter, ID19)
**Concerns about health**	*‘After quitting, it is much easier to breathe.’* ( Female, quitter, ID2) *‘My lung scan is clearer [after quitting].’* ( Female, quitter, ID3) *‘I don’t want to have all sorts of problems all over my body when I come out of prison.’* ( Male, quitter, ID10) *‘[During COVID-19] I worried about my health.’* ( Female, quitter, ID22) *‘My skin has become much whiter and cleaner.’* (Female, quitter, ID23)
**Money savings**	*‘[I quit] because cigarettes are expensive.’* ( Male, quitter, ID11) *‘I can save more money to buy snacks.’* ( Female, quitter, ID22) *‘A PIC’s salary is about HK$500–700 (about US$64–90) per month. It is not high … Participants would want the cash incentive for their families.’* ( Staff, ID27)

**Table 4:** Quit to Win (QTW) in prison: A trigger for behavior change identified by this qualitative study of people in custody in two Hong Kong prisons

*Major themes*	*Selected quotations*
**Health education**	*‘The frightening messages made me more conscious of the potential adverse consequences of smoking.’* (Female, quitter, ID5) *‘I saw that after the cigarette was inserted into the watermelon, the watermelon became a dark, tarry color, and I felt nauseous.’* ( Female, quitter, ID21)
**Quitting incentives**	*‘There was a cash reward when you participated, you could take photos with family, and meet your children and family members at the award ceremony.’* ( Female, quitter, ID1) *‘Getting a cash award was second in line; I participated mainly for my family.’* ( Male, quitter, ID8) *‘The QTW gave me a goal, and I wanted to see my family.’* ( Male, continuing smoker, ID14) *‘There are no additional visits, opening ceremony, and award ceremony this year. I truly want to see my family.’* (Female, quitter, ID26) *‘PIC could have an additional 4 special visits from families prior to COVID-19.’* ( Staff, ID27) *‘Visiting and encouragement from the “outside world” helped a lot in their quitting process. Some religious organizations outside and relatives used to visit them from time to time. However, most of them had stopped since the pandemic.* *Support for quitting is markedly reduced, which may hinder their quitting.’* ( Staff, ID28)
**Social support**	*Family:* *‘I stopped smoking after my mother visited me last time. She decided to quit together with me.’* ( Female, quitter, ID1) *‘My family encouraged me to join a smoking cessation class. After I joined the class, I learned about the QTW.’* (Female, quitter, ID3) *‘My family always encourage me to quit smoking.’* ( Female, quitter, ID25) *Peers:* *‘Smoking cessation courses were helpful in quitting. After attending courses with my friends, I did not smoke. I felt motivated.’* ( Female, quitter, ID2) *‘Having other people to quit together and encouraging each other made me more determined and gave me a goal. We would raise up our fists when we met to signal “add oil” to the other person.’* ( Male, quitter, ID10) *‘My friends didn't give me cigarettes after I joined QTW.’* ( Female, quitter, ID21) *Staff:* *‘Madam W was very supportive and helpful.’* ( Female, quitter, ID4) *‘I had less motivation on my own; the staff were truly supportive of me.’* ( Male, continuing smoker, ID9) *‘There was case sharing from a correctional staff member sharing his own quitting experience. The staff member spent extra private time to help us, which was very heart-warming.’* ( Male, quitter, ID17)

Based on the thematic analysis of the interview data, two main themes were identified: 1) quitting in prison: barriers and facilitators; and 2) QTW in prison: a trigger for behavior change.

### Quitting in prison: barriers and facilitators

*Stress, boredom, isolation, and lack of self-autonomy*

Stress and boredom were the main barriers to remaining abstinent from smoking in prison. PIC participants commonly experienced various adaptive challenges in adapting to the prison environment and used smoking as a coping mechanism for external stressors:

*‘I am truly unhappy [after imprisonment], so I smoke, but I know that smoking doesn’t solve the problem …’* (Female, continuing smoker, ID24)

PIC participants also felt bored due to the limited leisure activities in prison, and smoking helped to pass the time:

*‘Quitting in prison is harder than quitting outside because [inside] there are few things to do for leisure, so I smoked for leisure.’* (Female, quitter, ID1)

The suspension and reduction of social visits during the COVID-19 outbreak period made their lives more intolerable than before, and this worsened their sense of worry. PIC participants turned to smoking to relieve anxiety and boredom during this period of uncertainty and social isolation:

*‘PIC smoke during COVID-19 because they worry that they are abandoned by their family and wonder if their family doesn’t disclose facts to them.’* (Staff, ID27)

Lack of self-autonomy in prison decreased PIC participants’ self-efficacy in remaining abstinent. In response to the lack of autonomy in prison, a participant (male, quitter, ID17) who had not smoked before imprisonment said that he perceived smoking as a ‘symbol of freedom’ and that it was ‘relieving’ to smoke. When the participants were asked to predict the likelihood of relapse after the QTW, most predicted a higher risk of relapse because of the lack of autonomy in prison and a boring routine of prescribed activities:

*‘[Here] there is nothing we can do… Every day is repetitive, the same work every day …’* (Female, quitter, ID22)

*Nicotine dependence and lack of cessation medication*

PIC participants explained that quitting was difficult because of their nicotine dependence, especially for those who had co-occurring substance use disorders:

*‘I used to be addicted to alcohol, cocaine, and ketamine [before entering prison]. I got psychological cravings.’* (Female, quitter, ID5)

Correctional staff explained that many PIC had smoked since early adolescence, resulting in high nicotine dependence and difficulty in quitting:

*‘Many of the inmates started smoking at age 12–13 years. Some inmates have high nicotine dependence. They are willing to forgo two to three meals to smoke.’* (Staff, ID27)

Despite high nicotine dependence, PIC had limited access to nicotine replacement therapy (NRT):

*‘NRT is difficult to access. If needed, cessation medicines must be prescribed by doctors based on the PIC’s nicotine dependence. NRT patch doses are usually given for up to two weeks and then discontinued.’* (Staff, ID27)

*Barriers to moving to a different wing*

PIC had no choice on living in a smoking or smoke-free wing:

*‘It is decided by the Correctional Services Department's administration based on their medical and psychosocial history, health condition, and the availability and suitability of the jobs in the wing.’* (Staff, ID27)

A non-smoker or a smoker willing to quit might be assigned to the smoking wing. Moving from one wing to another would entail starting from the bottom of the job hierarchy with a lower salary and position:

*‘If I move to a smoke-free wing, I have to start over in a new job and at the lowest position.’* (Female, quitter, ID23)

Moreover, the job options in the smoke-free wing might not match their preferences:

*‘I don't like the work [in the smoke-free wing], so I would rather not move.’* (Female, quitter, ID21)

Therefore, moving to a smoke-free wing was not an easy option for PIC to quit smoking.

*Smoke-free wing*

Despite institutional barriers, some participants chose forced abstinence by moving to a smoke-free wing. Those who did so found that remaining abstinent was easier than before because cigarettes, triggers, and temptations were absent:

*‘[In a smoke-free wing] it is much easier for me to quit smoking because the temptation is relatively small.’* (Male, quitter, ID19)

In addition to avoiding relapses, the smoke-free wing had more entertainment time for watching TV than the smoking wing:

*‘I want to apply to move to a smoke-free wing, so that I will not relapse. In addition, the smoke-free wing gives us time to watch TV.’* (Female, quitter, ID5)

*Concerns about health*

PIC participants were aware of smoking-related harms:

*‘I didn’t want to have all sorts of problems all over my body when I came out of prison.’* (Male, quitter, ID10)

As smoking increases the risk for severe COVID-19 symptoms^20^, some participants reported a heightened perception of smoking hazards during the pandemic:

*‘[During COVID-19] I worried about my health.’* (Female, quitter, ID22)

*Money savings*

Financial constraints made PIC participants determined to quit because their earnings were extremely low compared to those in the outside world:

*‘A PIC’s salary is about HK*$*500–700 (about US*$*64–90) per month.’* (Staff, ID27)

The restricted incomes in prison made cigarettes less affordable:

*‘[I quit] because cigarettes are expensive.’* (Male, quitter, ID11)

PIC participants knew that they could save money by stopping smoking and thus ease the financial burden on their families.

### QTW in prison: a trigger for behavior change

*Health education*

Participants attended cessation courses during QTW when education slides, videos, and printed materials were provided. Health talks on the harms of smoking were increased to motivate participants to quit:

*‘The frightening messages made me more conscious of the potential adverse consequences of smoking.’* (Female, quitter, ID5)

Participants remembered the educational video that showed a watermelon turning black from the tar of burnt cigarettes. One participant expressed that the scene was so repulsive that it provoked a visceral reaction in her:

*‘I saw that after the cigarette was inserted into the watermelon, the watermelon became a dark, tarry color, and I felt nauseous.’* (Female, quitter, ID21)

*Quitting incentives*

Biochemically validated quitters of the QTW were awarded up to HK$1000 (about US$125) worth of cash coupons, an award ceremony was held to celebrate their abstinence, and they earned ‘privileges’ such as being granted extra social visits:

*‘There was a cash reward when you participated, you could take photos with family and met your children and family members at the award ceremony.’* (Female, quitter, ID1)

Facing prolonged family separation, many participants emphasized that the social visits and the award ceremony where they could physically meet their families were key drivers of their quitting success:

*‘Getting cash award was secondary; I participated mainly for my family.’* (Male, quitter, ID8)

However, since the COVID-19 outbreak, social visits have been suspended, and the award ceremony was canceled. Almost all participants interviewed in 2021 expressed dissatisfaction with the cancellations of these activities. Although the incentive of cash coupon remained, participants described that the strongest appeal of quitting (i.e. seeing family members) was lost:

*‘There are no additional visits, opening ceremony, and award ceremony this year. I really want to see my family.’* (Female, quitter, ID26)

Correctional staff echoed that the reduced outside support, through face-to-face means, had made quitting more difficult:

*‘Visiting and encouragement from “outside world” helped a lot in their quitting process … most of them have stopped since the pandemic. Support for quitting is markedly reduced, which may hinder their quitting.’* (Staff, ID28)

*Social support*

Support from family members, inmate peers, and correctional staff was a frequently mentioned trigger of quitting. PIC participants shared that their families consistently encouraged them to quit and to attend the smoking cessation course:

*‘I stopped smoking after my mother visited me last time. She decided to quit together with me.’* (Female, quitter, ID1)

Peer support also helped; PIC participants who joined QTW had developed a sense of solidarity:

*‘Having other people to quit together and encouraging each other made me more determined and gave me a goal. We would raise up our fists when we met to signal “add oil” to the other person.’* (Male, quitter, ID10)

The participants also mentioned having received support from correctional staff:

*‘I had less motivation on my own; the staff was truly supportive of me.’* (Male, continuing smoker, ID9)

A staff member sharing his own quitting story was also conducive to increasing PIC participants’ interests and making them feel cared:

*‘There was case sharing from a correctional staff member sharing his own quitting experience. The staff member spent extra private time to help us, which was very heart-warming.’* (Male, quitter, ID17)

## DISCUSSION

To our knowledge, this study is the first to introduce the QTW contest to prisons to promote quitting. We identified barriers (i.e. stress, boredom, isolation, lack of self-autonomy, nicotine dependence and lack of cessation medication, barriers to moving to a different wing) and facilitators (i.e. concerns about health, money savings, and the smoke-free wing) that impeded or supported smoking cessation during incarceration. QTW helped PIC overcome the barriers of quitting by serving as a trigger for behavior change. QTW provided education, support, and rewards that enhanced PIC’s awareness, motivation, and confidence to quit smoking.

We found that the social features of QTW facilitated quitting. First, QTW offered a golden opportunity to quit – a decision for behavioral change not only for PIC but also for their families and loved ones. To some extent, the PIC attempted to reduce the negative impact of their imprisonment on their families by demonstrating their potential to change for health. Second, QTW provided a supportive environment for quitting with special and attractive incentives for PIC. The participants felt warmth in the form of encouragement and support from family and correctional staff. Support from peers cultivated a sense of togetherness based on similar backgrounds and quitting experiences. Our study echoed the findings of prior research on the use of quitting peers to form close and supportive relationships with each other for smoking cessation^21^. Social visits provide PIC with opportunities to build outside communities, which may help to alleviate triggers of smoking, such as isolation and stress. Incentives tailored to PIC, such as social visits and the awards ceremony, are useful, particularly for those who have family ties or social bonds outside prison. Our results have shown that expanding QTW to other prisons and collaborating with correctional service agencies may benefit smoking PIC.

As the most important source of social support, family is a prominent facilitator of the decision to quit among PIC. Incarceration severs ties between PIC and their families and loved ones. For most PIC participants, the rewards of reuniting with their families for even a short while were more precious than cash rewards. In Chinese culture, prioritizing family values and filial piety are important to individuals^22^. A recent review found that family visits improved the mental well-being of PIC and reduced rule-breaking behaviors during incarceration^23^. Family contact and support can strengthen the bonds of family and society with PIC and cultivate pro-social feelings and behaviors^24^. Offering more incentives, such as increasing the number of family visits based on the duration of abstinence, has been suggested as a future intervention. Our results suggest that family contacts in various forms (e.g. prison visits, family days, phone calls, video calls, and mail) are effective incentives for smoking abstinence and other behavioral changes. However, non-smoking PIC may see this practice as unfair and may need similar incentives to make other positive behavioral changes.

Although the suspension of social visits disincentivized PIC to quit and sustain abstinence, we found that the COVID-19 outbreak raised awareness of smoking hazards and health among PIC. COVID-19 outbreaks are teachable moments to help PIC become motivated to change unhealthy habits (e.g. smoking, physical inactivity, and being overweight) and may make them more receptive to behavioral support. Health promotion activities, such as health talks on smoking and other factors that increase the risk of severe COVID-19 symptoms, might enhance health literacy and motivation for behavioral change among PIC.

At the individual level, stress, boredom, and isolation were identified as barriers to quitting in PIC, which was in line with findings in the general population^25,26^. Given the high prevalence of stress and mental illness in the prison population^4^, current cessation models and guidelines based on the general population may not adequately address their unique needs. Mental health counseling can be coupled with cessation support to equip PIC with skills for coping with stress. If made accessible to PIC, more relaxing activities, such as music, reading, and mindfulness meditation, can be adaptive strategies for addressing stress and isolation^27^.

At the institutional level, difficulties in switching to the smoke-free wing were barriers to quitting. When PIC are first admitted, safety and job suitability rather than smoking history are the priority concerns of wing assignment. Measures such as considering an individual’s smoking profile and intention to quit when making wing assignments, making more wings and work sites smoke-free, and maintaining the same salaries for PIC who switch to the smoke-free wing, may reduce such barriers. Another barrier is insufficient pharmacological support. Although NRT sampling can be prescribed to PIC who meet the treatment criteria, NRT is difficult to access due to concerns about its misuse. With careful assessment and monitoring, particularly for those who have a history of illicit drug use and high nicotine dependence, more NRT could be provided to those who would benefit from a few more doses.

### Limitations

Our study has several limitations. First, the study may have selection bias due to purposive sampling and recall bias from participants, which could affect the generalizability and accuracy of the findings. Second, we interviewed participants who were motivated to quit in a large smoking cessation contest. Our results cannot reveal all potential patterns and trajectories of quitting experiences in prisons. Larger quantitative studies can examine the full range of quitting and relapse experiences under usual circumstances. Third, security concerns precluded the audio recording of interviews. Although this restriction might have limited the richness of the participants’ descriptions of their quitting experiences, our scribes were able to capture the most salient experiences reported.

## CONCLUSIONS

We have provided qualitative findings on the barriers and facilitators of quitting smoking in prisons. Smoking cessation interventions targeting PIC should leverage social support, especially from family members and correctional staff, facilitate access to pharmacotherapy, and enhance stress-coping skills without smoking.

## Data Availability

The study protocol, statistical analysis plan, and de-identified data are available on reasonable request to the corresponding author.
